# The impact of imperfect screening tools on measuring the prevalence of epilepsy and headaches in Burkina Faso

**DOI:** 10.1371/journal.pntd.0007109

**Published:** 2019-01-17

**Authors:** Ida Sahlu, Cici Bauer, Rasmané Ganaba, Pierre-Marie Preux, Linda D. Cowan, Pierre Dorny, Athanase Millogo, Hélène Carabin

**Affiliations:** 1 Department of Epidemiology, Brown University School of Public Health, Providence, Rhode Island, United States of America; 2 Department of Biostatistics and Data Science, University of Texas Health Science Center at Houston, Texas, United States of America; 3 Agence de Formation de Recherche et d'Expertise en Santé pour l’Afrique (AFRICSanté), Bobo Dioulasso, Burkina Faso; 4 INSERM, Univ. Limoges, CHU Limoges, UMR 1094, Tropical Neuroepidemiology, Institute of Neuroepidemiology and Tropical Neurology, Limoges, France; 5 Department of Biostatistics and Epidemiology, College of Public Health, University of Oklahoma Health Sciences Center, Oklahoma City, Oklahoma, United States of America; 6 Unit of Veterinary Helminthology, Department of Biomedical Sciences, Institute of Tropical Medicine Antwerp, Antwerp, Belgium; 7 Department of Virology, Parasitology and Immunology, Faculty of Veterinary Medicine, Ghent University, Merelbeke, Belgium; 8 Departement of Internal Medicine, University Teaching Hospital, Bobo-Dioulasso, Burkina Faso; 9 Departement de Microbiologie et Pathologie, Faculté de Médecine Vétérinaire, Université de Montréal, Saint-Hyacinthe, Québec, Canada; Texas A&M University College Station, UNITED STATES

## Abstract

**Background:**

Epilepsy and progressively worsening severe chronic headaches (WSCH) are the two most common clinical manifestations of neurocysticercosis, a form of cysticercosis. Most community-based studies in sub-Saharan Africa (SSA) use a two-step approach (questionnaire and confirmation) to estimate the prevalence of these neurological disorders and neurocysticercosis. Few validate the questionnaire in the field or account for the imperfect nature of the screening questionnaire and the fact that only those who screen positive have the opportunity to be confirmed. This study aims to obtain community-based validity estimates of a screening questionnaire, and to assess the impact of verification bias and misclassification error on prevalence estimates of epilepsy and WSCH.

**Methodology/Principal findings:**

Baseline screening questionnaire followed by neurological examination data from a cluster randomized controlled trial collected between February 2011 and January 2012 were used. Bayesian latent-class models were applied to obtain verification bias adjusted validity estimates for the screening questionnaire. These models were also used to compare the adjusted prevalence estimates of epilepsy and WSCH to those directly obtained from the data (i.e. unadjusted prevalence estimates). Different priors were used and their corresponding posterior inference was compared for both WSCH and epilepsy. Screening data were available for 4768 individuals. For epilepsy, posterior estimates for the sensitivity varied with the priors used but remained robust for the specificity, with the highest estimates at 66.1% (95%BCI: 56.4%;75.3%) for sensitivity and 88.9% (88.0%;89.8%) for specificity. For WSCH, the sensitivity and specificity estimates remained robust, with the highest at 59.6% (49.7%;69.1%) and 88.6% (87.6%;89.6%), respectively. The unadjusted prevalence estimates were consistently lower than the adjusted prevalence estimates for both epilepsy and WSCH.

**Conclusions/Significance:**

This study demonstrates that in some settings, the prevalence of epilepsy and WSCH can be considerably underestimated when using the two-step approach. We provide an analytic solution to obtain more valid prevalence estimates of these neurological disorders, although more community-based validity studies are needed to reduce the uncertainty of the estimates. Valid estimates of these two neurological disorders are essential to obtain accurate burden values for neglected tropical diseases such as neurocysticercosis that manifest as epilepsy or WSCH.

**Trial registration:**

ClinicalTrials.gov NCT03095339.

## Author summary

Epilepsy and progressively worsening severe chronic headaches are the two most common clinical manifestations of neurocysticercosis, a form of cysticercosis. To understand where the prevalence of these neurological disorders is highest for targeted infection control, valid prevalence estimates are needed. Most neuroepidemiological studies conducted in low-resource settings use a two-step approach to identify cases (screening questionnaire followed by physician examination among screened positives) to obtain prevalence and burden estimates. We found that this most commonly-used two-step approach in community-based studies leads to an underestimation of the prevalence in our study. Our paper provides an analytic solution to reduce errors in estimating the prevalence of neurological disorders in community-based studies when using the two-step approach. Our proposed approach provides more valid estimates of the prevalence of these neurological disorders and could be used to better reflect the consequences that neglected tropical diseases manifesting as epilepsy or headaches have on people’s health and disabilities.

## Introduction

The Global Burden of Disease Study estimates epilepsy and migraines to be among the 30 leading causes of years of life lost due to disability [1]. The prevalence of epilepsy and headaches varies globally, likely due to different risk factors across populations or epidemiological biases [2,3]. A meta-analysis estimated the median prevalence of lifetime epilepsy in developing countries at 15.4‰ in rural and 10.3‰ in urban areas, higher than the estimated 5.8‰ in developed countries [4]. The estimated prevalence of lifetime epilepsy shows substantial variation within sub-Saharan Africa (SSA) [4–8], ranging from 7.3‰ to 29.5‰. In contrast, the prevalence of migraine in adults is reported to be higher in developed countries than that in developing countries, with an estimate of 15% in Europe and 5% in Africa [3]. The limited data collected on headaches in SSA suggest that the one-year period prevalence ranges from 3.0% to 5.4% for migraines, and 1.7% to 7.0% for tension-type headaches [9–11]. Reasons for the opposite trends in the frequency of epilepsy and headaches may be due to the distribution of socio-economical, cultural, and infectious risk factors and genetic susceptibilities, as well as important methodological differences between studies [1,3,12]. In particular, whereas prevalence data on epilepsy and headaches often originate from national surveys or nation-wide electronic health records in high income countries, it is not the case in SSA, where data originate from research projects conducted in a small number of communities. The way these neurological disorders are measured in different settings could result in important misclassification error bias which, in turn, could explain some of this variation in estimates.

Rural community-based studies in SSA often use a two-step approach to identify neurological disorder cases for prevalence estimates [13,14]. In step one, a screening test, often in the form of a questionnaire, is used to identify positive cases for the neurological disorder(s) of interest in the study population. The participants in this step can be randomly selected or identified via door-to-door visits. In step two, a physician or sometimes a neurologist confirms the given neurological disorder(s) through a medical examination, often only among those screened positive [15–19]. Diagnosis through electroencephalography is often not possible in rural community-based settings due to limited resources [13], and therefore, the physician/neurologist’s diagnostic is often considered as the gold standard.

The two-step approach is frequently used for studies in low-resource settings due to its convenience and cost-effectiveness. Despite its frequent use, the prevalence estimates from the two-step approach can be seriously biased from failure to account for the imperfect validity of the tests, referred to as misclassification error hereinafter, employed either at step one or step two, or both. The validity of a test is typically assessed by its sensitivity and specificity, and the correct sensitivity and specificity must be used to obtain unbiased prevalence estimates. However, for questionnaires used as the screening tool for epilepsy and headaches, especially those used in rural areas of SSA, little is known about their validity. In most studies, standardized screening questionnaires are used, but their validity is not determined in the study population [8–10,19]. To our knowledge, only two studies have reported validity estimates of epilepsy screening questionnaires in the general population; however, neither addresses potential verification bias in their validity estimates [20,21]. Verification bias occurs when the participants from step one have different probabilities of being selected for step two [22]. For example, individuals screened positive at step one have a much higher chance of being confirmed by a physician or neurologist, compared to those screened negative, who are rarely, if at all, examined at step two. As a result, the selected individuals at step two are not representative of the entire study population, which may lead to biased prevalence estimates.

These biases can have important consequences in evaluating the global burden of neglected tropical diseases. For example, epilepsy and progressively worsening severe chronic headaches (WSCH) are the most frequently observed clinical signs of neurocysticercosis (NCC), a preventable infection with the eggs of *Taenia solium* [23], which is present in communities with poor sanitation and free roaming pigs [24,25]. Most epidemiological studies evaluating the prevalence of NCC in communities will first identify people with epilepsy, and rarely with headaches, and invite them to obtain brain imaging to diagnose NCC. The proportion of NCC among people with epilepsy (or rarely headaches) is then used to estimate the prevalence of epilepsy-associated (or headaches-associated) NCC in the study population. Such estimates may then be combined to the prevalence of epilepsy or headaches in the population to estimate Disability Adjusted Life Years (DALYs) associated with NCC [26–28]. The global burden of disease initiative used this approach to estimate DALYs associated with cysticercosis [29]. Therefore, to obtain accurate estimates of the global burden of NCC, or of any neglected tropical disease causing neurological signs, we first need valid prevalence estimates of epilepsy and WSCH. From there, we may obtain more reliable assessments of the relative burden of NCC, or other neglected tropical diseases manifesting as epilepsy or headaches, compared to other infections or chronic diseases.

In this paper, we aimed to quantify the bias introduced in the prevalence estimates when failing to account for the verification bias and the imperfect validity of the screening tool using data collected from 60 villages in Burkina Faso. We also investigated the validity of screening questionnaire by estimating the sensitivity and specificity to detect epilepsy and WSCH.

## Methods

### Study setting and participants

Baseline cross-sectional data collected from February 2011 to January 2012 for a cluster randomized controlled trial were used. The aim of the parent study was to estimate the effectiveness of a community-based educational intervention to reduce the cumulative incidence of human and porcine cysticercosis in 60 villages of Burkina Faso [30]. From 70 to 80 individuals aged 5 years or above were sampled in each village using a cluster random sampling approach described elsewhere [30].

### Ethics

The University of Oklahoma Health Sciences Center Institutional Review Board and the Centre MURAZ ethical review panel (Burkina Faso) approved this study. The field staff read the written consent forms to participants and answered all their questions. Consent forms were signed, marked with a cross or a fingerprint by those who were unable to write. All consent forms were signed by a witness. Parents of individuals 5 to 16 years of age gave consent for their children. Individuals aged 10 to 15 years were invited to give their assent. All participants were given a bar of soap as incentive. The parent study was registered through clinicaltrials.gov (NCT03095339).

### Screening of epilepsy and severe chronic headaches

In step one of the two-step approach, each participant was screened for epilepsy and WSCH using a screening questionnaire (S1 Questionnaire). Questions related to epilepsy were based on the International League Against Epilepsy screening of epilepsy questionnaire developed by Preux *et al*.[31], and was previously used in three villages in the same study area [32]. Questions related to headaches were designed to capture NCC-related headaches [33].

### Diagnostic confirmation by physicians/neurologists

In step two, all individuals screened positive for either epilepsy or WSCH from the screening questionnaire were invited to be examined by a study physician. In addition, 231 screened-negative individuals were randomly selected to be examined by the physician. The medical examination results were collected on a medical examination questionnaire (S2 Questionnaire). Two medical examination rounds took place with the second round aimed at capturing individuals who were absent during the first round and to examine patients from the more remote province of Nayala. The physicians discussed any uncertain diagnosis with the neurologist on the phone at the time of the medical examination. At the end of the study, the neurologist reviewed all diagnoses and his final diagnosis was considered as the gold standard. The diagnostic result that confirmed whether the individual had epilepsy and/or WSCH was used to assess the validity of the screening questionnaire.

### Definition of epilepsy and severe chronic headaches

Epilepsy was defined as having more than one seizure of central nervous system origin without apparent cause [34]. Individuals not meeting the epilepsy case definition were considered as epilepsy-free (i.e., screened negative for epilepsy). Six individuals diagnosed with single epileptic seizures were excluded from all analyses.

WSCH was defined as having symptoms arising more than weekly for two weeks or more, with each episode lasting at least 3 hours, and progressively worsening in severity with time. Headaches had to be severe enough to require analgesic or to prohibit working, playing, attending school, or partaking in daily activities [35]. Individuals not meeting the WSCH definition were considered as WSCH-free (i.e., screened negative for WSCH).

### Statistical analysis

The statistical analyses aimed at assessing the bias from failing to correct for both verification bias and misclassification error, when estimating the prevalence of epilepsy and WSCH in the study population. To assess the degree of the bias, we first calculated the unadjusted estimates, where no correction was made for verification bias and misclassification error. The unadjusted estimates were obtained by running a Bayesian binomial model, where the number of confirmed cases of either epilepsy or WSCH was assumed to result from a binomial distribution with a probability corresponding to the unadjusted prevalence and the number of individuals screened. The prior choice for the prevalence parameter is discussed below.

The adjusted prevalence estimate was obtained by running a Bayesian latent-class model [22]. In this model, the probabilities that participants were selected for the confirmation test at step two were specified by a set of conditional distributions. Participants with different selection probabilities had different conditional probability distributions. This way, the selection probabilities were correctly accounted for, eliminating verification bias. To obtain the specificity and sensitivity estimates of the screening questionnaire while correcting for verification bias, we provided prior information on the model parameters of sensitivity and specificity. Different priors, including both informative and non-informative (i.e., vague) priors, were investigated in modeling either epilepsy or WSCH. Specifically, for epilepsy, one set of informative priors based on the sensitivity and specificity estimates for similar epilepsy screening questionnaires from two previous community-based studies [25, 26] were used. In these studies, sensitivity and specificity were estimated as 92.9% and 79.3%, and 99.6% and 72.4%, respectively. To allow some variability, the prior sensitivity and specificity values in our analysis were assumed to follow beta distributions with mean based on these validity estimates and a standard deviation of 0.05. This led to a Beta(54.8, 17.5) prior for sensitivity, and a Beta(12.9, 0.5) for specificity. We also considered the vague priors of Unif(0.5, 1) for both parameters. Since epilepsy was highly stigmatized in SSA [6] and some forms of partial epilepsy may be difficult to identify, we also used a vague prior of Unif(0.3, 1) for the sensitivity of epilepsy screening. Due to the lack of validity studies for WSCH screening, the same informative priors as epilepsy were adopted for WSCH. We also ran the model with vague priors of Unif(0.5, 1) for both the specificity and sensitivity, and results were compared with those obtained using informative priors.

When modeling each epilepsy and WSCH, we used a vague Unif(0, 0.3) for the unadjusted prevalence estimate. For the adjusted prevalence parameter, a vague prior of N(0, 10) was used (on logit scale).

We used WinBUGS [36] for all the Bayesian analyses and reported the posterior mean and 95% credible intervals. The bias was then evaluated by the ratio of the adjusted to the unadjusted estimates, with the Bayesian credible interval for the ratio excluding 1 indicating the existence of bias.

### Assessing the impact of different screening strategies on the bias introduced by verification bias and misclassification error

We examined three scenarios reflecting different screening strategies often used in community-based studies conducted in low-resource areas. In Scenario 1, we estimated the unadjusted prevalence using the screening information for only one neurological disorder instead of for epilepsy and WSCH, to reflect a common situation in the existing literature where only one neurological disorder was studied at a time instead of both disorders simultaneously. In Scenario 2, we estimated the unadjusted estimate using the information for participants that were only examined in the first medical round. This scenario was considered to reflect situations where there were insufficient personnel and monetary resources to find people absent from the village during the initial visit. Scenarios 1 and 2 are fairly common in field studies conducted in resource-poor contexts. In Scenario 3, we only used the screening information for the unadjusted prevalence estimate, reflecting a situation where the validity of the screening questionnaire cannot be assessed. We calculated the adjusted estimates resulting from each scenario, and evaluated the bias estimating the ratio of the verification bias and misclassification error-adjusted to the unadjusted estimates.

### Assessing the impact of the proposed methods on the frequency estimates of NCC-associated epilepsy and WSCH

In this exercise, the impact of verification bias and misclassification error on community-based estimates of NCC-associated epilepsy and WSCH prevalence and number of cases was evaluated. To obtain the estimated prevalence of NCC-associated epilepsy and WSCH for our study population, the adjusted and unadjusted estimates of epilepsy and WSCH prevalence were multiplied by the proportion of NCC among people with epilepsy reported in a meta-analysis [37] and the proportion of NCC among people with headaches reported in a case-control study [38]. We then investigated the difference between the adjusted and unadjusted prevalence estimates of NCC-associated epilepsy and WSCH. To obtain the difference between the number of NCC-associated epilepsy and WSCH cases in our study population, the estimated adjusted and unadjusted prevalence estimates were multiplied by the study population size. We assumed that the mean number of NCC-associated cases of epilepsy and WSCH would follow beta distributions with parameters based on the estimated means in the published studies (29% for epilepsy and 4.7% for headaches) [37,38] and a standard deviation of 2.75%. All estimates were run using the set of informative priors described earlier. The adjusted prevalence and number of cases of NCC-associated epilepsy and WSCH were also estimated under the three screening strategies described above.

## Results

### Description of the study population

**Fig 1:**
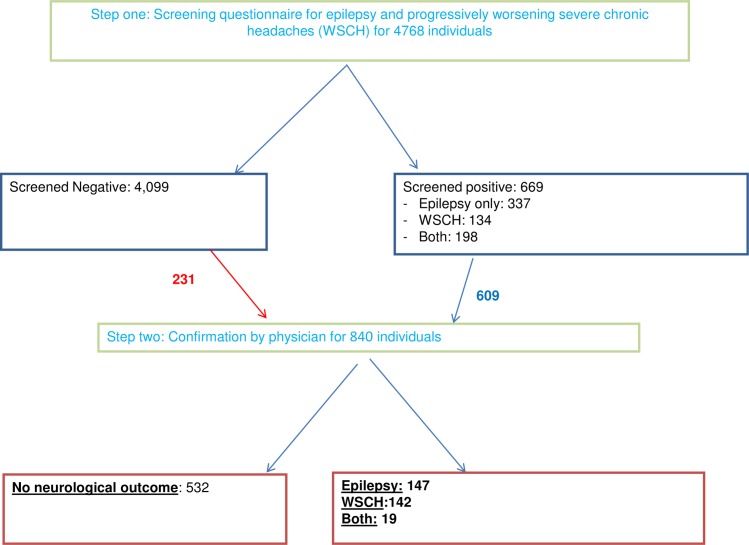
Flowchart of two-step confirmation for epilepsy and progressively worsening severe chronic headaches among 4768 individuals in 60 villages of Burkina Faso.

A total of 4794 individuals were sampled at baseline, including the analytical sample of 4768 with complete screening data. Of these, 669 (14.0%) screened positive for epilepsy (7.1%), WSCH (2.8%) or both (4.2%) (Table 1). A physician examined 609 (91.0%) screened-positive and 231 (5.6%) screened-negative individuals. The higher proportion of screened-positive examined by a physician compared to those screened-negative showed evidence for the need to adjust for potential verification bias (Fig 1).

**Table 1:** Characteristics of the 4768 individuals screened for epilepsy and worsening severe chronic headaches (WSCH) across 60 villages in three provinces of Burkina Faso, 2011–2012.

Characteristic	N (%)
**Age (24 missing)**	
6–17 years	1515 (31.9%)
18–40 years	1679 (35.4%)
41 years and older	1550 (32.7%)
**Gender (1 missing)**	
Female	2564 (53.8%)
Male	2203 (46.2%)
**Education**	
Did not complete primary school	4171 (87.5%)
Completed primary school	371 (7.8%)
Completed at least secondary school	226 (4.7%)
**Occupation (1 missing)**	
Student/Pupil	924 (19.4%)
Farmer/gardener	1819 (38.2%)
Housewife/house cleaner	1672 (35.0%)
Commerce/ Salaried/ Skilled self-employed/ Unemployed	352 (7.4%)
**Screening results**	
Negative	4099 (86.0%)
Positive for epilepsy	337 (7.0%)
Positive for WSCH	134 (2.8%)
Positive for epilepsy and WSCH	198 (4.2%)
**Diagnosis results from medical exam**	
None	4460 (93.5%)
Epilepsy	147 (3.1%)
WSCH	142 (3.0%)
Epilepsy and WSCH	19 (0.4%)

Impact of verification bias and misclassification error on the prevalence estimates of epilepsy and WSCH

Of those examined in step two, 748, 57 and 35 were seen at the first, second and both medical rounds, respectively. Perfect agreement was observed for those examined at both rounds. Table 1 describes the characteristics of the analytical sample, and the screening and medical examination results. The majority of participants were either a farmer or housewife and a high proportion did not complete primary school.

Table 2 provides the posterior estimates of the unadjusted prevalence and the corresponding adjusted prevalence with the associated sensitivity and specificity estimates, using the different sets of priors. The epilepsy screening questionnaire showed posterior sensitivity and specificity estimates of moderate variation with different priors used, while the specificity estimates remained robust. The posterior sensitivity estimate was 44.7% (95%BCI: 33.0%;60.0%) with the most vague prior and increased to 66.1% (95%BCI: 56.4%,75.3%) with the informative priors based on previous literature. Posterior sensitivity and specificity estimates of the WSCH screening questionnaire were less affected by the priors. Despite the variation in the estimated sensitivity and specificity, the unadjusted prevalence estimates were consistently lower than the adjusted ones for both epilepsy and WSCH, as indicated by the posterior bias distribution lying under the value of 1. Somewhat less bias was observed using the informative priors.

**Table 2:** Unadjusted and adjusted prevalence estimates (95% Bayesian Credible Interval (95%BCI)) of lifetime epilepsy and severe chronic headaches (WSCH) using data from 4768 individuals in Boulkiemdé, Nayala and Sanguié provinces, Burkina Faso. The table shows results from different priors used for sensitivity, specificity and prevalence as well as posterior estimates of the sensitivity and specificity of the screening questionnaire for epilepsy and WSCH using these priors. All analyses used the prior of N(0, 10) for the prevalence parameter.

	Posterior Estimate (95%BCI) for Epilepsy^a^	Posterior Estimate (95%BCI) for WSCH^a^
Unadjusted prevalence	3.5% (3.0%;4.0%)	3.4% (2.9%;3.9%)
**Vague priors set 1:** Sensitivity Uniform(0.3,1) and Specificity Uniform(0.5,1)		
Adjusted prevalence	7.9% (5.9%;10.6%)	Not applicable
Bias^b^	0.4 (0.3;0.6)	Not applicable
Sensitivity	44.7% (33.0%;60.0%)	Not applicable
Specificity	88.6% (87.6%;89.6%)	Not applicable
**Vague prior set 2:** Sensitivity Uniform(0.5,1) and Specificity Uniform(0.5,1)		
Adjusted prevalence	6.7% (5.4%;8.0%)	6.7% (5.6%;7.9%)
Bias^b^	0.5 (0.4;0.7)	0.5 (0.4;0.6)
Sensitivity	53.7% (50.2%;64.7%)	51.7% (50.1%;58.1%)
Specificity	88.8% (87.8%;89.7%)	88.4% (87.4%;89.3%)
**Informative prior:** Sensitivity ^c^Beta(54.8, 17.5) and Specificity ^d^Beta(12.9, 0.5)		
Adjusted prevalence	5.6% (4.6%;6.8%)	6.0% (4.9%;7.4%)
Bias^b^	0.6 (0.5;0.8)	0.6 (0.4;0.7)
Sensitivity	66.1% (56.4%;75.3%)	59.6% (49.7%;69.1%)
Specificity	88.9% (88.0%;89.8%)	88.6% (87.6%;89.6%)

^a^Includes 19 individuals with both epilepsy and WSCH

^b^Bias was estimated as follows: Unadjusted prevalence/ Adjusted prevalence

^c^Beta(54.8, 17.5): mean = 0.759, variance = 0.0025

^d^Beta(12.9, 0.5): mean = 0. 963, variance = 0.0025

**Fig 2:**
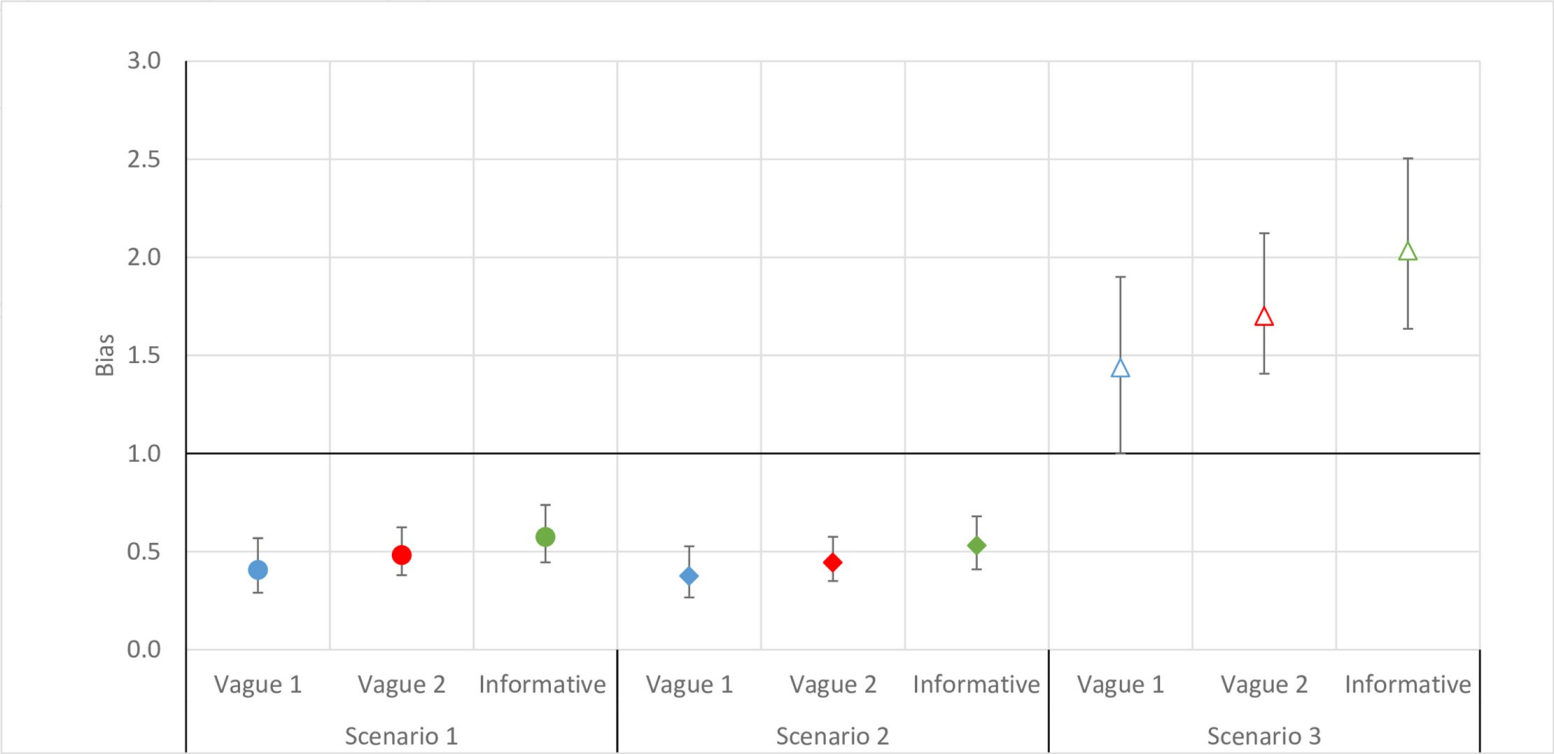
Bias (unadjusted prevalence/adjusted prevalence) observed from estimating epilepsy prevalence under different scenarios among 4768 individuals across 60 villages in three provinces of Burkina Faso, 2011–2012. Blue: Vague prior for sensitivity (Uniform(0.3,1)) and specificity (Uniform(0.5,1)), Red: Vague prior for sensitivity and specificity (both Uniform(0.5,1) and Green: Informative prior for sensitivity (Beta(54.8,17.5)) and specificity (Beta(12.9,0.5)). Scenario 1: the unadjusted prevalence estimated using the screening information for only one neurological disorder. Scenario 2: the unadjusted prevalence estimated using only the confirmation information from the first medical round. Scenario 3: the unadjusted prevalence estimated in the situation where a medical examination was not conducted.

**Fig 3:**
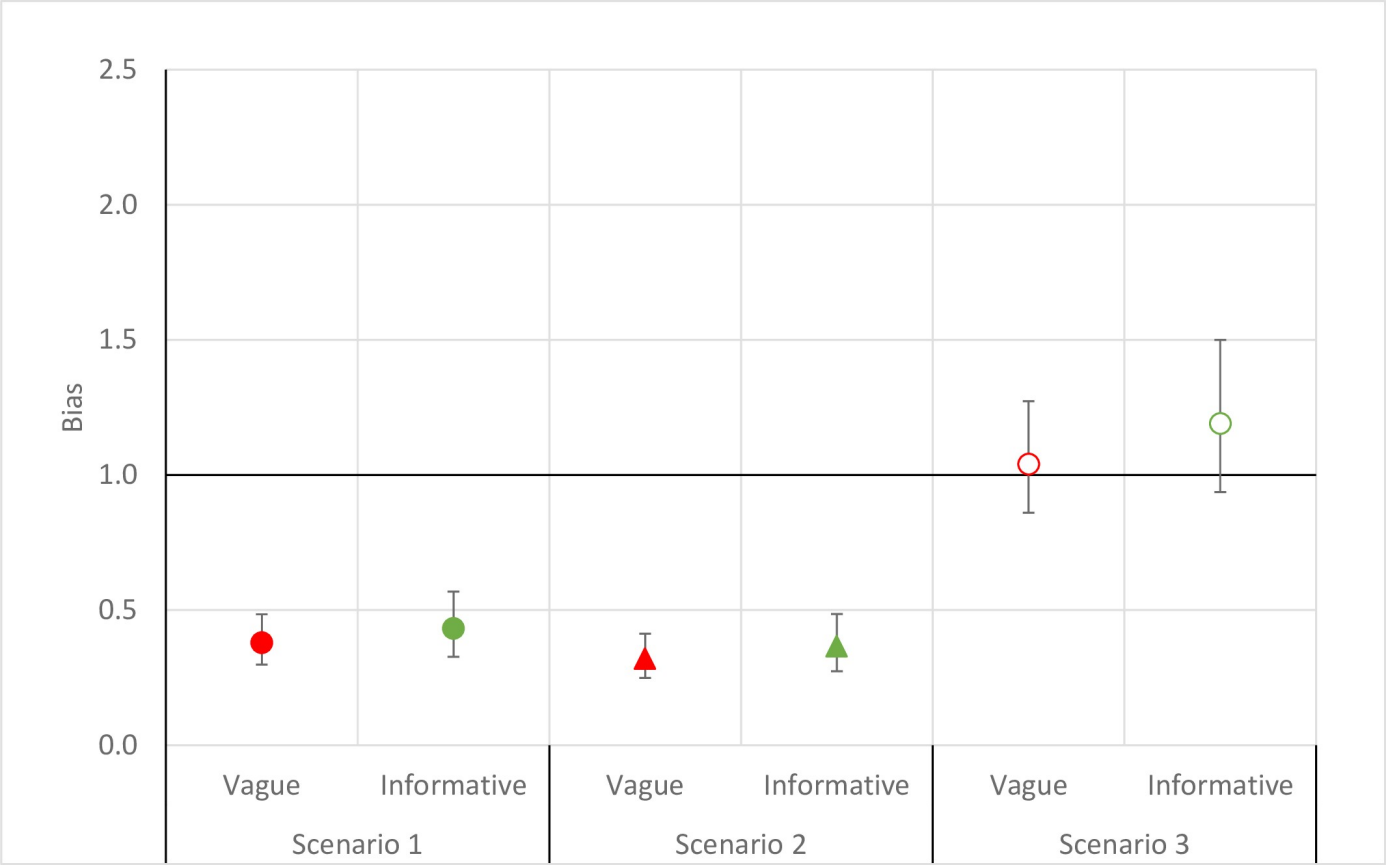
Bias (unadjusted prevalence/adjusted prevalence) observed from estimating severe chronic headaches prevalence under different scenarios among 4768 individuals across 60 villages in three provinces of Burkina Faso, 2011–2012. Red: Vague prior for sensitivity and specificity (both Uniform(0.5,1) and Green: Informative prior for sensitivity (Beta(54.8,17.5)) and specificity (Beta(12.9,0.50)). Scenario 1: the unadjusted prevalence estimated using the screening information for only one neurological disorder. Scenario 2: the unadjusted prevalence estimated using only the confirmation information from the first medical round. Scenario 3: the unadjusted prevalence estimated in the situation where a medical examination was not conducted.

Bias (i.e., where the unadjusted estimate was smaller than the adjusted estimate) was introduced for both epilepsy and WSCH (Figs 2 and 3) under the two most common screening strategies found in the literature, namely Scenarios 1 (i.e. when we assumed that the study would only screen for one neurological disorder) and 2 (i.e. when resources would not allow for returning to communities to examine those absent during a first visit). The magnitude of the bias was similar to that observed in the main analyses for epilepsy, but more marked for WSCH. For Scenario 3 (i.e. when only screening results were used), an important positive bias was present where the unadjusted prevalence estimate of 11.2% (95%BCI: 10.4%; 12.2%) was considerably larger than the adjusted prevalence for epilepsy when more informative priors were used. For WSCH, Scenario 3 resulted in a negligible positive bias. The magnitude of the bias did not vary extensively by the priors used for epilepsy and WSCH, except for epilepsy in Scenario 3.

### Impact of verification bias and misclassification error on the frequency estimates of NCC-associated epilepsy and WSCH in the base scenario and under different screening strategies

Using the study data, the differences between the adjusted and unadjusted prevalence and number of NCC-associated epilepsy were 0.6% (95%BCI: 0.3%; 1.0%) and 29 cases (95%BCI: 13; 49), respectively.

## Discussion

This was the first study to estimate the sensitivity and specificity of a screening questionnaire for epilepsy and WSCH in the community while adjusting for both verification and misclassification error bias. This was also the first study to quantify the bias from failing to account for verification and misclassification error bias when estimating the prevalence of epilepsy and WSCH in a community-based study.

Our sampling strategy was not designed to provide population prevalence estimates of epilepsy and WSCH in Burkina Faso as a whole, in the three study provinces, or even the villages selected for the parent study. Indeed, our sampling strategy, which favored concessions with pigs, was likely to result in higher estimates of prevalences than what would have been observed if a simple random sampling strategy had been adopted. Nonetheless, we provided below prevalence estimates from other community-based studies conducted in resource-poor settings using the two-step approach. In our study sample, the unadjusted prevalence of epilepsy was higher than that of 1.6% (95%CI: 1.2%;2.0%), 1.1% (95%CI: 0.9%; 1.4%) and 0.5% (95%CI: 0.2%;0.8%) found in Benin, Tanzania and Nigeria, respectively [7,15,39]. Our estimate was also higher than that of 0.6% (95%CI: 0.5%;0.7%) found in Cambodia where a screening questionnaire similar to ours was used [40]. Similarly, our unadjusted estimate was higher than the first study conducted in rural Burkina Faso, which estimated an epilepsy prevalence of 1.1% in 18 villages [41]. Our prevalence estimate was most similar to a recent Burkinabé study, conducted by the same research group, which estimated a lifetime epilepsy prevalence of 4.5% (95%CI: 3.3%;6.0%) in three villages purposely sampled to have a high prevalence of epilepsy [32]. This supported the suspicion that our study sample might represent people at higher risk of epilepsy than the general population.

Our unadjusted estimate of lifetime WSCH prevalence was similar to the lifetime migraine prevalence estimate of 3.3% (95%CI: 2.4%;4.6%) in Benin [42], and lower than the lifetime migraine prevalence estimate of 5.3% (95%CI: 5.0%; 5.6%) in Nigeria [43]. Lifetime prevalence estimates of tension-type headaches in SSA were unavailable for comparison. Compared to published one-year prevalence estimates of tension-type headaches, ours was higher than the 1.7% (95%CI: 1.5%; 1.9%) prevalence in Ethiopia among adults 20 years and older, and lower than the 7.0% (95%CI: 6.5%;7.6%) prevalence in Tanzania among all ages [9,11]. This suggested that our sampling strategy might have not influenced the estimated frequency of WSCH as much as it did for epilepsy.

The posterior estimates of the specificity for the neurological screening questionnaire were similar for epilepsy and WSCH, and were consistently around 88%. These estimates were slightly lower than those previously reported [20,21], possibly because our study population had a higher proportion of false negatives compared to the previously published validity studies. Study participants in our study may also have been less likely to report their symptoms compared to those in Ecuador and Bolivia, perhaps due to the stigmatizing effects of epilepsy in SSA [6]. The posterior estimates of sensitivity varied depending on the priors used, particularly for epilepsy. The sensitivity posterior median was higher when prior knowledge [20,21] information was used and lower for vague priors; although the credible intervals overlapped. A similar observation where the sensitivity parameter was more affected to the prior choice was also found in a study assessing the validity measures of a screening test for human papillomavirus [44].

Our sensitivity estimates were lower and specificity estimates higher for epilepsy than that found in validation studies conducted in clinical settings with estimates of sensitivity between 91% and 100% and specificity between 51% and 85% [19,20,45]. Placencia *et al*. found lower sensitivity and similar specificity estimates when the same screening questionnaire was used in the community as compared to the clinic [20]. Such discrepancies may be due to spectrum bias where populations in clinical settings have more severe disease and acknowledge their symptoms more, thereby increasing the test sensitivity. The observed estimates from clinical settings may also result in part from verification bias, which typically leads to an overestimation of the sensitivity and underestimation of the specificity [22]. Our sensitivity estimates for epilepsy were similar to the two other community-based studies with sensitivities of 72.4% (95% CI: 52.8–87.3) and 79.3% [20,21]. To our knowledge, prevalence studies using the two-step approach for WSCH have not reported validity estimates.

Even in a situation where 90% of those screened positive were examined by a physician, significant biases were observed. The unadjusted prevalence estimates were consistently lower than the adjusted prevalence estimates regardless of the priors used. However, the adjusted prevalence estimates were highly dependent on priors, particularly for epilepsy, which introduced considerable uncertainty. Such uncertainty could be reduced by conducting more community studies assessing and reporting validity estimates of screening. The validity estimates were expected to vary from one community to the next, due to how epilepsy and WSCH were reported by patients and to the way interviewers were trained to use the questionnaire. This would also result in varied bias estimates. Therefore, the reported sensitivity and specificity estimates of the screening questionnaire may not be applicable to other communities.

We chose the Bayesian framework [46,47] to simultaneously correct for verification bias and misclassification error in the two-step approach for two reasons. First, verification bias can be treated as a missing data problem, and therefore be corrected in a straightforward manner. Second, verification bias and misclassification error can be addressed simultaneously by including an additional level in the Bayesian model estimating the specificity and sensitivity of the invalid test(s). The capture-recapture method is an alternative approach for obtaining corrected prevalence estimates [13]. This method combines multiple sources of information independently, such as medical records and non-medical interviews with community members, with the two-step approach. This method yielded higher prevalence estimates than the two-step approach alone in two studies in Benin [7,42]. Our bias estimation method using their data yielded similar results (between 0.5 and 0.3). However, the capture-recapture method has multiple difficult-to-meet assumptions: closed population, statistical independence between sources, identical case definitions across multiple sources and requires more personnel resources [7,42]. Our study was less resource-intensive and provided an alternative to the capture-recapture method.

When screening strategies commonly used in community-based studies were explored, the unadjusted prevalence was lower than the adjusted prevalence in the two most frequently encountered scenarios, and the bias estimates were similar to that observed under the screening strategy used in the main analysis of this study. In our study, we had resources to capture those missed through the first medical round and we screened for two neurological outcomes, which resulted in more individuals being confirmed in step two compared to most community-based studies. Despite this more complete examination of individuals screened positive, the level of bias was similar to that estimated for screening strategies commonly used in community-based studies. In the last scenario explored, the prevalence relied entirely on the screening questionnaire, which led to a large number of false positive cases of epilepsy. This was especially true because the prevalence of epilepsy was relatively low, and hence, there were relatively more people without epilepsy who were false positives than people with epilepsy who were false negatives, even if the screening test’s specificity was much better than its sensitivity. As opposed to the other scenarios, failure to use physician confirmation led to an overestimate of the adjusted prevalence of epilepsy. This scenario could occur when economical and personnel resources are too limited for physician confirmation. For WSCH, the estimated negative bias (i.e. when the unadjusted estimate was lower than the adjusted estimate) was more marked in the two scenarios most often encountered in community-based studies than when using the screening strategy in the main analysis of this study. This suggested that by only screening for WSCH with or without two rounds, more WSCH cases were missed. There is rising evidence that seizures might increase the risk of headaches [48,49]. Hence, it is possible that screening for both outcomes improved the detection of WSCH through the neurological examination of people screening positive for epilepsy. The opposite (i.e. the screening of people with WSCH increases the detection of epilepsy) might not be as marked in our study because a lot less participants screened positive for WSCH than for epilepsy or both epilepsy and WSCH. In the last scenario where the prevalence estimate relied solely on the screening questionnaire, we observed negligible positive bias for WSCH. This finding illustrated that screening for both epilepsy and WSCH led to a less biased estimate of the prevalence compared to the more commonly used approach of only screening for one neurological condition. This was not observed for the last scenario for epilepsy because screening for WSCH along with epilepsy might not increase the detection of epilepsy.

When we examined the impact of verification and misclassification bias on the estimation of the prevalence and frequency of NCC-associated epilepsy and WSCH, we found that the prevalence and number of NCC were underestimated when using two-step approach regardless of the scenario. In the scenario without confirmation by a physician or neurologist, we found that the number of NCC-associated epilepsy cases were over-estimated, while we did not observe a difference between the unadjusted and adjusted prevalence and number of NCC-associated WSCH cases. These results could have important consequences in the estimation of the monetary and non-monetary burden of NCC locally and globally. For example, the estimated global DALYs of NCC-associated epilepsy were estimated to be 2,788,426 (95% Uncertainty Estimates (UI): 2,137,613–3,606,582) by the Foodborne Epidemiology Research Group in 2010 [50] and to be 468,100 (95% UI: 322,900–625,800) in 2016 by the Global Burden of Disease 2016 Collaborative [51]. Moreover, the 2015 DALYs for migraine were estimated to be 32,899,000 (95%UI: 20,295,000–48,945,000) [52]. If these estimates used underlying NCC-associated epilepsy and headaches frequencies which were underestimated to a similar level as in our study, it could have important consequences on how NCC would rank among all diseases locally and globally and on policy making. Indeed, if the burden of NCC were higher than currently believed, more resources should be allocated to control it.

Our study has several limitations. First, the physicians examined a small proportion of individuals screened negative. Increasing this proportion would have reduced the variance of our posterior sensitivity estimates. Second, our study used two medical rounds with different physicians. However, perfect agreement between the two physicians was observed and all diagnoses were reviewed and confirmed by the neurologist, minimizing the possibility for bias. Third, we could not conclude that our prevalence estimates for the study population were reflective of the prevalence for the villages due to the sampling scheme. Since the aim of our study was to quantify the bias that rose from failure to account for misclassification error and verification biases by comparing the unadjusted estimates to the adjusted estimates, we believe our findings were still of importance.

Our results suggest that the burden of epilepsy and WSCH in low-resource settings might be much higher than previously reported. Future studies should consider using the statistical models presented here to account for the imperfect nature of screening questionnaires. Bias-adjusted prevalence estimates of these two neurological disorders will improve our understanding of the burden of these conditions and help identify where cysticercosis may be present. More valid prevalence estimates will allow for the development of targeted cysticercosis control programs in those communities.

## Supporting information

S1 Checklist STROBE checklist. (DOC)

S1 Questionnaire English version of the screening questionnaire for severe chronic headaches and epilepsy. (DOCX)

S2 Questionnaire English version of the medical examination questionnaire for severe chronic headaches and epilepsy. (DOC)

S1 Dataset Table with individual variables: Village, province, age category, gender, education completed, occupation, screening result, physician confirmation and result. (XLS)
