# Supplementary material for: The impact of imperfect screening tools on measuring the prevalence of epilepsy and headaches in Burkina Faso
Source: PLoS Negl Trop Dis. 2019 Jan 17;13(1):e0007109. doi: 10.1371/journal.pntd.0007109 (PMC6353216; doi:10.1371/journal.pntd.0007109)
Supplement: S1 Questionnaire — (DOCX) [file pntd.0007109.s002.docx]

S1 Questionnaire: English version of screening questionnaire for severe chronic headaches and epilepsy.

Severe Chronic Headaches:

1. Have you ever had bad headaches that did not go away and that got worse over time?
2. Were these headaches bad enough to keep you from doing your daily chores, work or going to school?

Epilepsy

3) Have you ever had any of the following?

a. Sudden loss of consciousness and episodes of incontinence or foaming of the mouth or tongue biting?

b. A brief period of absence(s) or loss(es) of contact with the surroundings that starts suddenly?

b.1. How often has this happened?

c. Uncontrollable twitching or jerking or abnormal movements of one or more limb(s) (convulsions) that starts suddenly and lasts for a period of a few minutes?

c.1. How often has this happened?

d. Sudden onset of a brief period of hearing or smelling or seeing things that are not there or feeling strange body sensations?

e. Were you ever told that you had epilepsy or that you had had an epileptic seizure?

f. Have you ever had seizures or fits?

[***If ‘yes’ or ‘can’t remember/don’t know’ to any of the questions – this person noted to be examined by the field doctor***]
