# Supplementary material for: The impact of imperfect screening tools on measuring the prevalence of epilepsy and headaches in Burkina Faso
Source: PLoS Negl Trop Dis. 2019 Jan 17;13(1):e0007109. doi: 10.1371/journal.pntd.0007109 (PMC6353216; doi:10.1371/journal.pntd.0007109)
Supplement: S2 Questionnaire — (DOC) [file pntd.0007109.s003.doc]

##### ÉFÉ CAB

***Improving pig management to prevent epilepsy in Burkina Faso***

Centre Hospitalier Universitaire Souro Sanou, AFRICSanté & University of Oklahoma Health Sciences Center

# NEUROLOGICAL HISTORY AND EXAMINATION

Last Name : _______________________ First Name: _____________________________

Questionnaire number :________

Individual identification number : **|___|___|___|___|___|___|___|___|___|___|**

Name of the physician ___________________________________

Household number _____________________________________

Village number _________________________________________________

**Epilepsy and seizures**

1. Describe in detail what happens to the patient when they have a seizure:

_____________________________________________________________________________

_____________________________________________________________________________

_____________________________________________________________________________

_____________________________________________________________________________

_____________________________________________________________________________

_____________________________________________________________________________

2. Do the patient’s seizures occur ONLY when the following situations are present? [*Check all that apply*]

🞎 Febrile convulsions 🞎 Alcohol intake 🞎 Malaria 🞎 Medical drugs

🞎 Eclampsia 🞎 Other (Specify) ___________________________________________

🞎 **NO**, the seizures happen for no reason

🞎 Does not know, not sure

3. Are the seizures started by any of the following events? [*Check all that apply*]

🞎 Emotion 🞎 Alcohol intake 🞎 Sleep 🞎 Lack of sleep

🞎 Light stimulation *(sun reflecting on water, television, disco)* 🞎 Hyperventilation

🞎 Menstruation 🞎 Stopping of antiepileptic treatment 🞎 Drugs or toxic agents

🞎 When wakes up or in the following hour

🞎 The seizures sometimes start for no reason

🞎 The seizures NEVER start following any of these events

🞎 Other (Specify) _________________________________________________

__________________________________________________________________________

🞎 Does not know, not sure

4. Has the patient ever had two or more seizures which occurred more than 24 hours apart?

🞎 Yes 🞎 No 🞎 Does not know

5. Has the patient ever had [*check all that apply*]

🞎 Generalized tonic-clonic seizures 🞎 Myoclonic generalized seizures

🞎 Atonic generalized seizures? 🞎 Tonic seizures 🞎 Absences

🞎 Other types of generalized seizures (Specify) ___________________________________________

🞎 Simple partial seizures 🞎 Complex partial seizures

🞎 Partial seizures secondary generalized 🞎 Other type of seizures difficult to classify

🞎 *Status epilepticus* 🞎 Does not know

6. How many seizures has the patient had in the past 12 months? ___________________________

🞎 Has not had a seizure in the past 12 months [Skip to 9] 🞎 Does not know

6.1 What type of seizures were they? [*Check all that apply*]

🞎 Generalized tonic-clonic seizures 🞎 Myoclonic generalized seizures

🞎 Atonic generalized seizures 🞎 Tonic seizures 🞎 Absences

🞎 Other types of generalized seizures (Specify) ____________________________________

🞎 Simple partial seizures 🞎 Complex partial seizures

🞎 Partial seizures secondary generalized 🞎 Other type of seizures difficult to classify

🞎 *Status epilepticus* 🞎 Does not know

7. How many seizures has the patient had in the past 6 months? ___________________________

🞎 Has not had a seizure in the past 6 months [Skip to 9] 🞎 Does not know

8. How many seizures has the patient had in the past month (4 weeks)? _______________________

🞎 Has not had a seizure in the past month [Skip to 9] 🞎 Does not know

9. How old was the patient at the first seizure?

🞎 During the first 10 days of life 🞎 More than 10 days to 6 months of age

🞎 More than 6 months to 2 years of age 🞎 More than 2 years to 6 years of age

🞎 More than 6 years to 12 years of age 🞎 More than 12 years to 20 years of age

🞎 More than 20 years to 40 years of age 🞎 More than 40 years of age

🞎 Does not know

10. Period of time between the last seizure and the date of this neurological examination?

🞎 Less than 24 hours 🞎 1 to 30 days 🞎 More than 30 days to 1 year

🞎 More than 1 year to 3 years 🞎 More than 3 years to 5 years

🞎 More than 5 years 🞎 Does not know

11.1 Is this patient’s history consistent with a diagnosis of a single seizure?

🞎 Definite 🞎 Probable 🞎 No 🞎 Uncertain

[**NOTE ON PDA: any person with Definite or Probable – read and invite to sign the CT scan consent from invite to a CT scan. Note that this person should be put on the list to get the scan. Any person with an uncertain should be discussed with Prof. Millogo**]

11.2 Is this patient’s history consistent with a diagnosis of epilepsy (i.e., recurrent, unprovoked seizures)?

🞎 Definite 🞎 Probable 🞎 No 🞎 Uncertain

[**NOTE ON PDA: any person with Definite or Probable – read and invite to sign the CT scan consent from invite to a CT scan. Note that this person should be put on the list to get the scan. Any person with an uncertain should be discussed with Prof. Millogo**]

**Severe progressive headache**

12. Headache pain sufficient to interfere with activities of daily life or requires analgesics?

🞎 Yes 🞎 No 🞎 Does not know

13. Headache frequency?

🞎 Generally constant 🞎 Daily 🞎 Weekly

🞎 Monthly [*Skip to Q18*] 🞎 Less than once per month [*Skip to Q18*]

🞎 Unable to determine [*Skip to Q18*]

14. What type of pain are the headaches usually accompanied with?

🞎 Throbbing (pulsating)

🞎 Piercing (perforating sensation)

🞎 Stabbing or sharp

🞎 Pressure

15. How do the headaches occur?

🞎 They are continuous 🞎 Begin suddenly and uexpectedly (paroxysmal)

🞎 They occur periodically (episodic)

16. Have headaches become **progressively worse since they started,** have they remained at the same level of pain, or have they gotten less painful?

🞎 More severe 🞎 About the same ¨Better 🞎 Uncertain

17. When did headaches begin? 🞎 Within past month 🞎 Within 6 months

🞎 Within past year 🞎 More than one year ago 🞎 Uncertain

18. How long do the headaches usually last?

🞎 Several minutes but less than 1 hour 🞎 From 1 to 2 hours 🞎 From 3 to 6 hours 🞎 Half a day

🞎 All day 🞎 Other (Specify ___________________)

19. Are the headaches triggered by any of the following situation?

🞎1 Yes 🞎1 No [*Go to question 20*]

19.1 What are they triggered by?

🞎 Brushing teeth 🞎 Shaving

🞎 Drinking alcohol 🞎 Chewing

🞎 Other (Specify __________________________________________________)

20. Are the headaches usually accompanied by [Check all that apply]:

🞎 Nausea 🞎 Vomiting 🞎 Photophobia 🞎 Phonophobia

🞎 Impossible to determine/ does not know

21. Just before or during headaches does the patient have visual disturbance?

🞎 Yes 🞎 No [*Go to question 22*]

21.1 What are those visual disturbances (check all that apply)

🞎 Black spots 🞎 Gaps in visual field 🞎 Zig-zag lines 🞎 Other evidence of aura

🞎 Other visual disturbance (describe) _________________ 🞎 Cannot determine

22. Which picture below best shows how much pain you have when you have headaches?

23. Location of headache: (*Show the patient the illustration of the cranium and ask them to identify all the places where they feel pain. Mark on the illustration accordingly.)*

23.1 Do the headaches tend to start on one particular side before moving to the next?

🞎 Yes 🞎 No [*Go to Q23*]

23.2 On which side does the headaches usually start?

🞎 Left 🞎 Right

24. Brief description of headaches:

_____________________________________________________________________________

_____________________________________________________________________________

_____________________________________________________________________________

_____________________________________________________________________________

25. Is this patient’s history consistent with a diagnosis of severe, progressively worsening headaches?

🞎 Definite 🞎 Probable 🞎 No 🞎 Uncertain

[**NOTE ON PDA: Anyone with a « Definite » or « Probable » -- invite to a CT scan of the brain with the CT scan consent form. Anyone with an «uncertain» -- discuss with Millogo**]

**Neurological examination**

26. Mental status (assess while completing the exam):

🞎 Alert and awake 🞎 Confusion

🞎 Drowsiness 🞎 Stupor 🞎 Coma

🞎 Other (specify _____________________________)

27. Cranial Nerves 🞎 Not assessed [*Skip to Q28*]

27.1 Gross visual acuity: 🞎 both eyes normal 🞎 either eye abnormal 🞎 not assessed

27.2 Cranial nerves III, IV, VI 🞎 normal 🞎 abnormal 🞎 not assessed

27.3 Cranial nerve V 🞎 normal 🞎 abnormal 🞎 not assessed

27.4 Cranial nerve VII 🞎 normal 🞎 abnormal 🞎 not assessed

27.5 Cranial nerve VIII 🞎 normal 🞎 abnormal 🞎 not assessed

27.6 Cranial nerves IX, X 🞎 normal 🞎 abnormal 🞎 not assessed

27.7 Cranial nerve XI 🞎 normal 🞎 abnormal 🞎 not assessed

27.8 Cranial nerve XII 🞎 normal 🞎 abnormal 🞎 not assessed

28. Muscle tone

🞎 Abnormal 🞎 Normal [*Skip to Q29*] 🞎 Not assessed [*Skip to Q29*]

28.1 If abnormal, indicate which limb(s): 🞎 right arm 🞎 left arm 🞎 right leg 🞎 left leg

28.2 If abnormal, indicate type of abnormality: 🞎 hypertonia 🞎 hypotonia

29. Power in main muscle groups 🞎 normal 🞎 abnormal 🞎 not assessed

29.1 If abnormal, which limb(s): 🞎 right arm 🞎 left arm 🞎 right leg 🞎 left leg

30. Coordination 🞎 normal 🞎 abnormal 🞎 not assessed

31. Tendon reflexes 🞎 normal 🞎 abnormal 🞎 not assessed

31.1 If abnormal, indicate type of abnormality: 🞎 hyperreflexia 🞎 hyporeflexia

32. Plantar responses 🞎 normal 🞎 abnormal 🞎 not assessed

33. Sensation (pin prick) 🞎 normal 🞎 abnormal 🞎 not assessed

32.1 If abnormal, indicate which limb(s): 🞎 right arm 🞎 left arm 🞎 right leg 🞎 left leg

34. Vibration sense 🞎 normal 🞎 abnormal 🞎 not assessed

34.1 If abnormal, indicate which limb(s): 🞎 right arm 🞎 left arm 🞎 right leg 🞎 left leg

35. Gait 🞎 normal 🞎 abnormal 🞎 not assessed

36. Romberg’s test 🞎 normal 🞎 abnormal 🞎 not assessed

37. Overall general neurological assessment:

🞎 Normal 🞎 Definitely abnormal (hard signs) 🞎 Equivocally abnormal (soft signs)

37.1 If abnormal, location of condition: 🞎 brain 🞎 spinal cord

🞎 peripheral nervous system🞎 muscles

37.2 If abnormal, is condition? 🞎 focal 🞎 multifocal 🞎 system-wide

37.3 If abnormal, most likely diagnosis? ___________________________________________

_____________________________________________________________________

**Physical examination**

38. Presence of burns or scars from burns? 🞎 Yes 🞎 No

38.1 If Yes, specify __________________________________________________

39. Presence of fracture or previous fracture? 🞎 Yes 🞎 No

39.1 If Yes, specify __________________________________________________

40. Other trauma? 🞎 Yes specify __________________________________🞎 No

41. Has the patient ever had or been treated for tuberculosis?

🞎 Yes 🞎 No 🞎 Does not know

42. Does the patient show sub-cutaneous nodules associated with cysticercosis [*take a picture of the lesion with the consent of the patient*]? 🞎 Yes 🞎 No

42.1. If yes, how many nodules are there? _______________________________

42.2 If yes, where are they located ? ____________________________________

___________________________________________________________________________
